# Prostate cancer lesion detection, volume quantification and high-grade cancer differentiation using cancer risk maps derived from multiparametric MRI with histopathology as the reference standard

**DOI:** 10.1016/j.mri.2023.01.006

**Published:** 2023-01-11

**Authors:** Matthew Gibbons, Jeffry P. Simko, Peter R. Carroll, Susan M. Noworolski

**Affiliations:** a Department of Radiology and Biomedical Imaging, University of California, San Francisco, CA, United States; b Department of Urology, University of California, San Francisco, CA, United States; c Department of Pathology, University of California, San Francisco, CA, United States

**Keywords:** Prostate cancer, Multiparametric MRI, Histopathology, Diffusion-weighted imaging, Dynamic contrast-enhanced imaging

## Abstract

Multi-parametric MRI (mpMRI) has proven itself a clinically useful tool to assess prostate cancer (PCa). Our objective was to generate PCa risk maps to quantify the volume and location of both all PCa and high grade (Gleason grade group ≥ 3) PCa. Such capabilities would aid physicians and patients in treatment decisions, targeting biopsy, and planning focal therapy. A cohort of men with biopsy proven prostate cancer and pre-prostatectomy mpMRI were studied. PCa and benign ROIs (1524) were identified on mpMRI and histopathology with histopathology serving as the reference standard. Logistic regression models were created to differentiate PCa from benign tissues. The MRI images were registered to ensure correct overlay. The cancer models were applied to each image voxel within prostates to create probability maps of cancer and of high-grade cancer. Use of an optimum probability threshold quantified PCa volume for all lesions >0.1 cc. Accuracies were calculated using area under the curve (AUC) for the receiver operating characteristic (ROC). The PCa models utilized apparent diffusion coefficient (ADC), T2 weighted (T2W), dynamic contrast-enhanced MRI (DCE MRI) enhancement slope, and DCE MRI washout as the statistically significant MRI scans. Application of the PCa maps method provided total PCa volume and individual lesion volumes. The AUCs derived from lesion analysis were 0.91 for all PCa and 0.73 for high-grade PCa. At the optimum threshold, the PCa maps detected 135 / 150 (90%) histopathological lesions >0.1 cc. This study showed the feasibility of cancer risk maps, created from pre-prostatectomy, mpMR images validated with histopathology, to detect PCa lesions >0.1 cc. The method quantified the volume of cancer within the prostate. Method improvements were identified by determining root causes for over and underestimation of cancer volumes. The maps have the potential for improved non-invasive capability in quantitative detection, localization, volume estimation, and MRI characterization of PCa.

## 1. Introduction

The prevalence of PCa results in high absolute mortality [1]. However, treatment side effects can be significant and must be balanced against what can be slow and low risk progression of disease [2]. Improvements in diagnostic methods remain necessary to accurately differentiate cases of indolent disease from cases with high progression risk to optimize patient care and minimize unnecessary treatment. The objective of this study was to quantify and automate lesion identification, including volume and severity, by generating PCa maps from mpMRI scans. These properties combined with mean MRI parameters within identified MRI lesions could be used to develop mpMRI biomarkers to assist with PCa identification, predictions of PCa progression, and improved targeting of biopsy or focal therapy.

Clinical practice has been improved by combining biopsy and multi-parametric magnetic resonance imaging (mpMRI). Biopsy remains the clinical standard with its determination of disease severity through tumor grading (Gleason grade group (GG)) [3]. Diagnosis and assessment of prostate cancer (PCa) is enhanced using mpMRI with its ability to detect, locate, and determine the extent of PCa with the added benefit of being non-invasive [4–6]. Together the two techniques mitigate inadequacies of each applied separately. Biopsy samples only a small fraction of the prostate and may miss instances of significant disease [7,8]. MRI encompasses the whole prostate and can assist in targeting biopsy [4,9]. Despite implementation of the Prostate Imaging Reporting and Data System (PI-RADS), mpMRI remains a qualitative assessment which suffers from variability due to differences in radiologist skill and experience [10]. If mpMRI can be made more quantitative, targeting of biopsy and treatment as well as decisions on active surveillance versus treatment could be improved.

To improve and automate the detection of PCa using mpMRI, machine learning and artificial intelligence methods have been developed [11]. Approaches for identification of PCa include multivariate regression [12,13] and convolutional neural networks [14,15]. The “ground truth” used to train and test models has included radiologist PI-RADS interpretation of mpMRI [16], histopathology of targeted biopsy [15], and histopathology of entirely embedded prostate glands after prostatectomy [12,17]. In this study, we combined strict requirements (identify individual lesion location, volume, and severity down to small size with histopathology as the reference standard) with an objective of improved accuracy metrics. Multivariate logistic regression utilizing MRI parameters was implemented to create models for categorizing PCa. Histopathology after prostatectomy was the reference standard. mpMRI image sets were prepared by registration and normalization to improve the consistency and accuracy of the model results [18]. Cancer risk maps were generated applying the models to prostate voxels within the mpMRI images. Analysis was performed to determine whether the mpMRI cancer risk maps could detect and locate PCa lesions 0.1 cc and larger. We also assessed the capability to quantify lesion volume and Gleason grade group 3 and higher versus lower Gleason grade groups as validated by histopathology.

The maps have the potential for improved non-invasive capability in quantitative detection, localization, and volume estimation of PCa. Such capabilities would aid physicians and patients in treatment decisions, targeting biopsy, and planning focal therapy.

## 2. Materials and methods

### 2.1. Overview

Several steps were necessary to generate cancer maps and lesion masks from mpMRI images and histopathological data. MRI images were registered to the T2W images so mpMRI parameters were properly overlaid. The PCa models were generated in a manner similar to prior studies [18,19]. The mpMRI intensities of clear-cut prostate tissues, representing a small percentage of the prostate volumes, were identified on MRI and histopathology. These regions of interest (ROIs) were characterized and tabulated. Logistic regression was implemented on the data set with MRI parameters as factors and histology as ground truth creating the models to differentiate anterior fibromuscular stroma (AFMS), other benign tissues, low-grade cancer, and high-grade cancer in the peripheral zone (PZ) and the transitional zone (TZ). Prostates were segmented into PZ and TZ. The cancer models were applied to each image voxel within the prostate creating cancer probability maps. From the maps, cancer lesion masks were generated by imposing a threshold to binarize the probability maps. The masks identified the location and volume of lesions.

### 2.2. Study population

The cohort included patients with untreated, biopsy-proven prostate cancer who had provided written research consent, underwent prostatectomy within one year of mpMRI exam, and had subsequent whole-mount histopathology evaluation of the excised prostate gland. Of the 78 patients remaining in the cohort, five were excluded for severe MRI artifacts or a significant portion of the prostate missing from MRI or histopathology. This yielded a study population of seventy-three participants [18,19]. The Institutional Review Board approved the study and the methods met Health Insurance Portability and Accountability Act compliance.

### 2.3. MR imaging

Images or maps resulting from T2-weighted imaging (T2W), diffusion weighted imaging (DWI), and dynamic contrast-enhanced MR imaging (DCE MRI) contributed to the cancer models. Scanning was performed on 3 T systems (GE Healthcare, Chicago, IL, USA) utilizing both an external phased array coil and an endorectal coil (MedRad, Bayer HealthCare LLC, Whippany, NJ). The T2W images were corrected [20,21] to remove non-uniformity across the field-of-view (FOV) caused by the reception profile of the endorectal coil. Image slices were acquired in the oblique axial plane [22].

The Fast Spin Echo (FSE) T2-weighted images had parameters: TR/TE 6000/96 ms, FOV 18 cm × 18 cm, matrix 512 × 512, slice thickness 3 mm, Nex = 1. Diffusion weighted images were scanned with a 2D single-shot echo-planar imaging spin echo sequence with parameters: TR/TE 4000/78–90 ms, b-value 0 and 600 s/mm^2^, slice thickness 3 mm. Scans had either a conventional FOV 24 cm × 24 cm (matrix 128 × 128, Nex = 4) or reduced FOV 18 cm × 9 cm (matrix 128 × 64, Nex = 6) with the latter providing susceptibility artifacts reduction [22]. In-house software calculated apparent diffusion coefficient (ADC) and fractional anisotropy (FA) maps. A 3D fast Spoiled Gradient Recalled (SPGR) sequence was used for DCE MRI with parameters: TR/TE 3.5/0.9 ms, flip angle 5°, FOV 26 cm × 26 cm (matrix 256 × 256), slice thickness 3 mm, time 5 min, time resolution 6 to 13 s. The contrast agent was gadopentetate dimeglumine (Gd-DTPA) (Magnevist; Bayer, Whippany, NJ). Contrast enhancement was monitored with maps of peak enhancement (PE), maximum enhancement slope (ES), and washout rate (WO). Quantities were calculated for each voxel [23] to create the maps.

### 2.4. MR image normalization

T2W and DCE MRI images were normalized to decrease the variance from patient to patient. For T2W, a scaling factor was determined by averaging over two ROIs selected within the obturator muscles next to the prostate. The normalized T2W for each voxel, *i*, for a patient, j, is given by:

(3) $$T2W_{\text{norm\_}i}=\frac{T2W_{i}}{T2W_{\text{muscle\_}j}}$$

The DCE MRI images were scaled based on the peak enhancement, *PE*_*ave*_*j*_, averaged over the prostate for each case, *j*, and the average PE over all cases, *PE*_*ave*_. This form was used because analysis of prostate tissue characteristics indicated only small difference in PE between prostate tissues [18], but sometimes significant differences between patients. The normalization equations for DCE MRI ES and DCE MRI WO for each voxel, *i*, were:

(4) $$ES_{\text{norm}\_i}=ES_{i}•\left(\frac{PE_{\text{ave}}}{PE_{\text{ave}\_j}}\right)$$

(5) $$WO_{\text{norm\_}i}=WO_{i}•\left(\frac{PE_{\text{ave}}}{PE_{\text{ave}\_j}}\right)$$

### 2.5. Histopathology and regions of interest

Analogous to prior studies [18,19], histology of entirely embedded prostatectomy specimens cut at 3 mm slices as whole mount sections were evaluated, with areas of tumor (including Gleason score), benign glands, stroma and inflammation marked as histology ROIs. Histology ROI location information from the slides was translated to the MRI images by manually drawing MRI ROIs onto the T2W images based on anatomical landmarks with the consensus of two readers. The mean values of MRI parameters within the ROIs were tabulated to provide MRI parameter distributions associated with the tissue types identified for the corresponding histology ROIs. For this study the tissue types used were benign tissue, AFMS, PCa Gleason grade group 2 and lower (Gleason score (GS) ≤ 3 + 4), and PCa Gleason grade group 3 and higher (GS > 3 + 4). This data was used as input to the cancer models.

### 2.6. Cancer models

Logistic regression with the MRI parameters as factors was implemented to create models for distinguishing tissue types [18]. JMP software was used for logistic regression [24]. The logistic regression models of the mpMRI parameters had the form shown in Eq. (6) for the probability, ℙ, and Eq. (7) for the multivariate log-odds function, *t*, with *n* mpMRI parameters, *x*.


(6) ℙ=1/(1+exp(−t))

(7) $$t=A+\sum_{i=1}^{n}B_{i}x_{i}+\sum_{j=1}^{n}C_{j}x_{j}^{2}$$

The logistic regression analysis was preceded by a mixed, stepwise algorithm where factors were added or removed from the fit depending on their significance [24]. In this analysis the criterion for parameter inclusion was a *p*-value ≤0.15. Once complete, the fits were manually checked for overfitting. If the fit dependency for a factor was inconsistent with the dependencies indicated by comparison of the univariate means, the factor was removed from consideration and the fit recomputed.

Three model types were generated: a) AFMS versus cancer, b) cancer versus benign (not including AFMS) and c) Grade Group ≤ 2 versus Grade Group ≥ 3. For each type, separate models were calculated for the TZ and the PZ. As shown in previous work [18], AFMS has MRI characteristics distinct from other prostate tissues. Because of this, it was useful to generate a separate model to exclude AFMS.

Area under the curve (AUC) of receiver operating characteristic (ROC) curves were used to evaluate the logistic regression fits. Sensitivity and specificity were reported at the point on the ROC curve closest to equal sensitivity and specificity, and sensitivity - (1 - specificity) at a maximum. This weights false negatives and false positives in a similar manner. To assess the performance of each model, a 4-fold cross-validation was performed. The AUC mean and 95% confidence interval were calculated.

### 2.7. Image registration and segmentation

The images were aligned using automatic rigid registration. This task is more complicated for images of the prostate than, for example, the brain. Challenges include: a) images spanning the body interior, which do not include the body’s surface to aid registration, b) a FOV wide in-plane, but thin out-of-plane (aspect ratio of 4:1 in the superior to inferior direction), c) some images are calculated maps with noise in low signal regions, such as the rectum, and d) anatomic deformations due to the physical impact of the probe [25]. There are also large contrast differences between image types. A registration algorithm was devised to be stable despite the large aspect ratio of the image domain. The algorithm had two steps: a) a 2D in-plane registration of the entire FOV, followed by b) a 3D registration localized to the prostate. In the registration steps a normalized mutual information registration technique was implemented using Matlab [26]. Since the purpose of this study was to characterize the efficacy of the cancer maps as opposed to fully automated registration, the automated registration results were checked manually. Registration error criteria were set as 2 mm for in-plane (1/3 the diameter of a 0.1 cc lesion), and 3 mm for out-of-plane (the slice thickness). Images failing the criteria were manually corrected.

The cancer models for TZ and PZ must be applied only in the corresponding zones of the prostate. To facilitate this requirement, the TZ and PZ were manually segmented in the T2W image domain. With all mpMRI images registered and interpolated to the T2W image space, the prostate segmentations were used to directly mask the images for the desired zone in the prostate.

### 2.8. Cancer maps

**Fig. 1.**
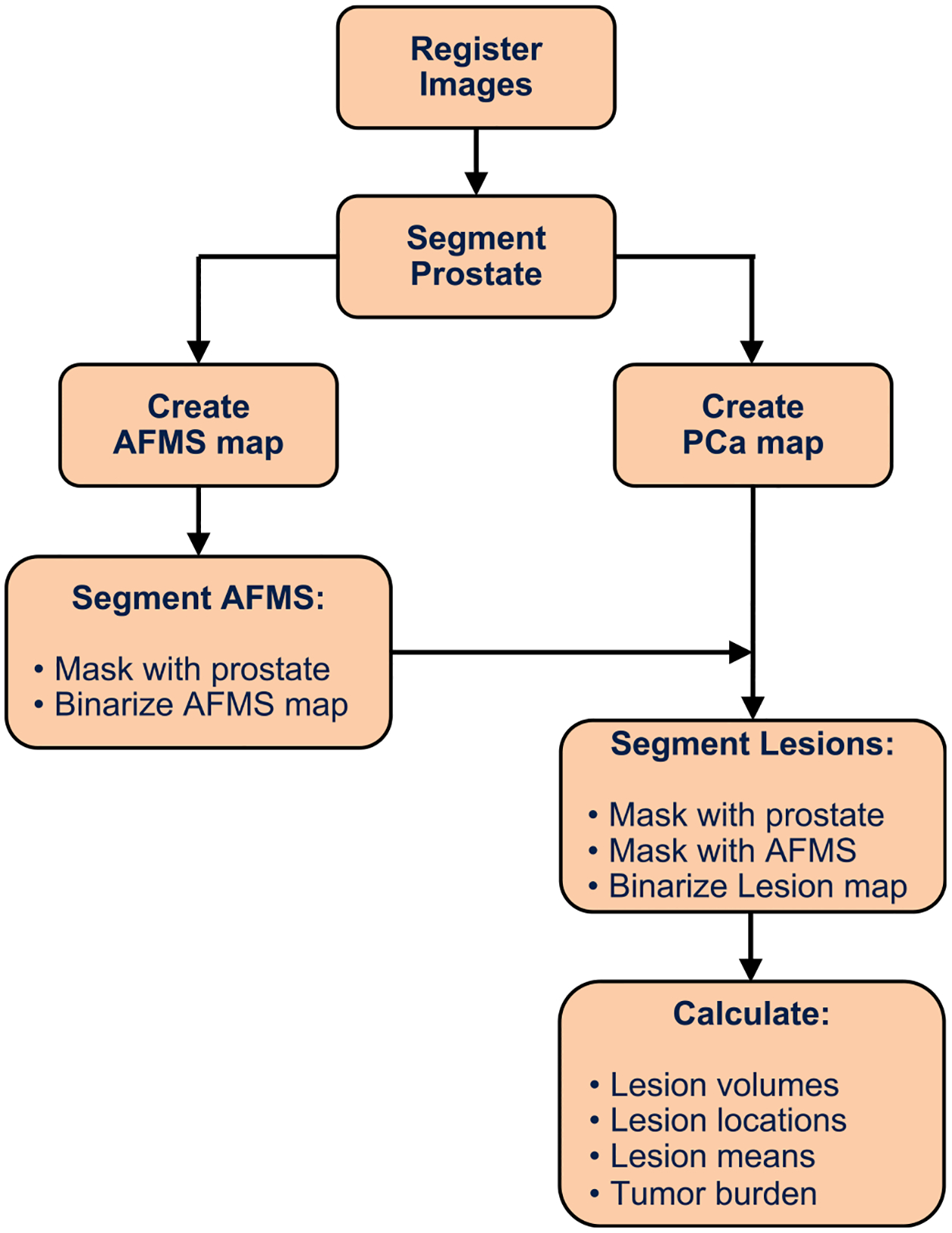
Prostate cancer maps flowchart.

The cancer maps were generated in several steps. First, the AFMS model was applied to each voxel to identify the AFMS and similar benign stromal tissues. Those voxels with an AFMS probability threshold of ≥50% were removed from the prostate segmentation mask effectively defining them as not PCa. Subsequently, the cancer models for TZ (or PZ) were applied on a voxel-by-voxel basis to the images. The maps were limited to voxels within the prostate by masking the image with the AFMS modified prostate segmentation for the TZ (or PZ). The MRI tumor burden, defined as the total volume of cancer, was calculated at several PCa probability thresholds by calculating the volume of voxels above each threshold. The ratio of MRI cancer volume to histopathology cancer volume was then determined at each threshold for each patient. From this result a cohort mean ratio was calculated for each threshold. The threshold for binarizing the PCa maps into PCa masks was determined where the volume ratio had a cohort mean of one. Two different thresholds were found for the two models (PCa versus benign and PCa GG 1&2 versus PCa GG 3–5). An additional filter was applied for voxels to be designated as high-grade (PCa GG 3–5). So, the high-grade PCa, voxels were required to have probability above threshold for both PCa models. In other words, high-grade voxels were confirmed to be cancer, not benign tissue, and then confirmed to be high-grade not low grade. Fig. 1 provides a flowchart for the method.

The MRI tumor burden was analyzed by several methods. MRI PCa volume was plotted versus histopathology PCa volume of each case to assess deviation from a 1:1 linear trend. Bounding lines, symmetric about the 1:1 line, were defined to indicate outliers as (4/3) • *volume*_*pathology*_ + 1 *cc* and (3/4) • (*volume*_*pathology*_ −1 *cc*). The bounding lines are consistent with the definitions for the lesion confusion matrix factors. Bland-Altman analyses were performed to test the correlation between the MRI and histology PCa volumes. A binary classification was performed with the factors given in Table 1 to determine sensitivity and specificity of the maps to classify the cancer and benign volumes. The factors are defined to categorize MRI PCa volume ≤ histology PCa volume as true positive (TP) and MRI benign volume ≤ histology benign volume as true negative (TN).

The matching of individual lesions was also assessed. Since application of a risk threshold converted the probability maps into binary lesion masks, individual MRI lesions were defined as separate objects composed of connected cancer voxels within each 3D binary image. MRI lesion centroids were automatically located within the prostate and reported in sections defined by splits along three axes anterior/mid/posterior, left/mid/right, and apex/mid/base. We shall use the term HGROI for high-grade PCa objects since they may not have been separate lesions but only part of a lesion having both low- and high-grade cancer.

**Fig. 2.**
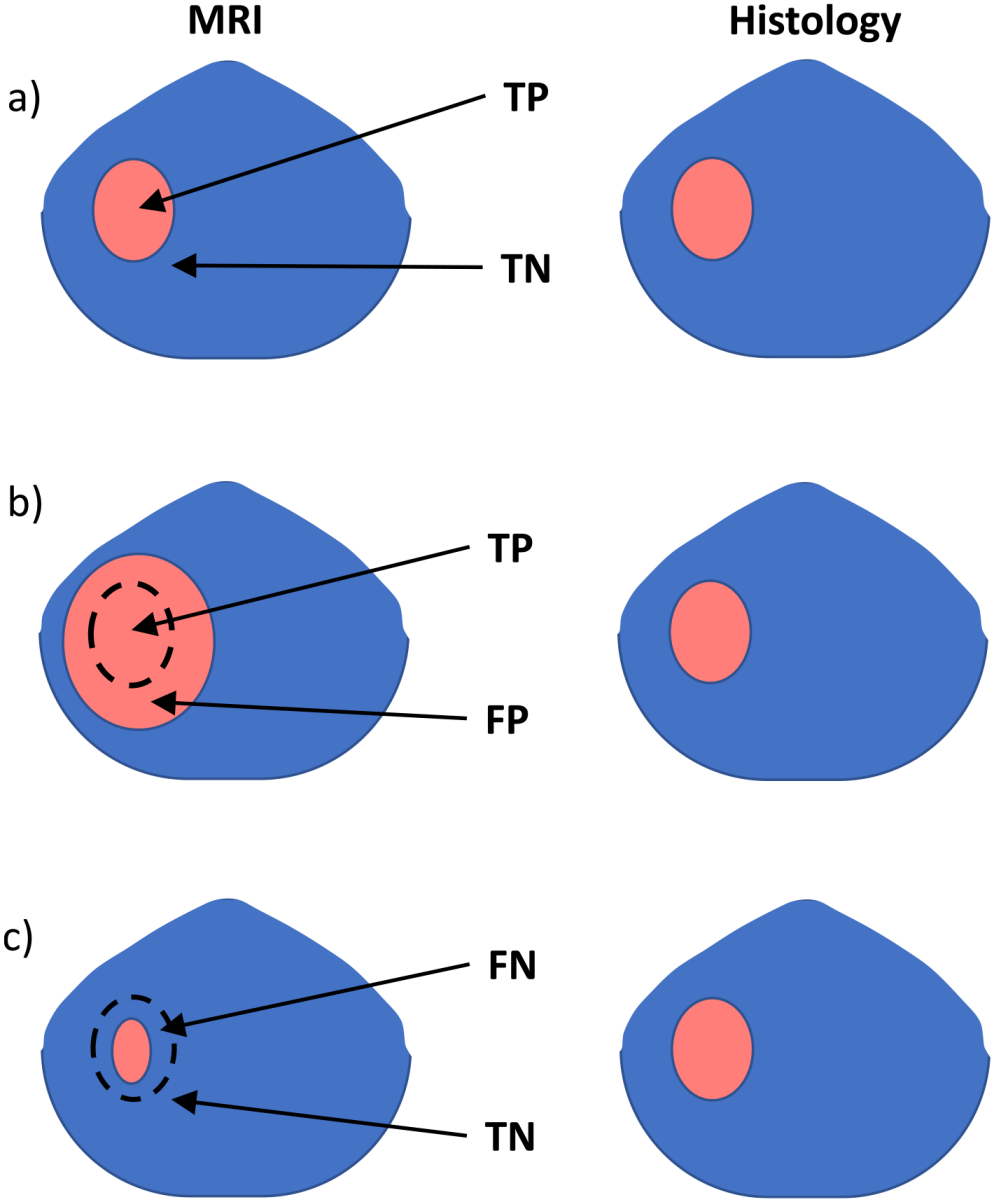
Examples of MRI lesion volume versus histology lesion volume with different classification results: a) TP when the MRI lesion size is within the outlier limits. b) FP when the MRI lesion size is larger than 1.33 • *volume*_*pathology*_ + 1 *cc*. c) FN when the MRI lesion volume is <0.75 • (*volumepathology* − 1 *cc*).

Lesions and HGROIs >0.1 cc were identified and manually compared to histopathology. Co-located lesions between MRI and histopathology were matched. Binary classification was performed to determine the sensitivity and specificity. The categories for individual lesion matching are defined in Table 2. Fig. 2 provides examples of MRI lesion size affecting classification. Causes for errors in lesion identification and cancer volumes were identified by manual review of the images and maps.

## 3. Results

### 3.1. Cohort demographics

The demographics of the participants were as follows: mean age 64.1 ± 6.1 years, median PSA 6.2 ng/ml (Q1 = 4.3 ng/ml, Q3 = 8.8 ng/ml), median prostatectomy Gleason score 7 (Q1 = 6, Q3 = 8). Given the inclusion criteria, the median time between MRI scan and prostatectomy was 38 days (Q1 = 14 days, Q3 = 76.8 days).

### 3.2. Registration

As confirmed by visual inspection, automated registration increased the cohort alignment yield from 82% to 95%. Cases failing alignment were manually corrected. For comparison, the cohort characteristics of the observed pathology lesions are listed in Table 3. The median in-plane diameter of lesions identified on histopathology was 6 mm, while the minimum lesion diameter was 2 mm.

### 3.3. Cancer models

The logistic regression models were generated based on the mpMRI characteristics of 1524 tissue ROIs (111 AFMS, 1016 other benign, and 397 PCa). There were 668 ROIs in the TZ and 856 ROIs in the PZ. The logistic regression coefficients for the cancer models as well as additional details on the tissue ROIs are listed in the supplemental information. The AUC of the AFMS models were 0.996 and 0.986 for the PZ and TZ, respectively. The AFMS models were derived without 4-fold cross validation since an insufficient number of AFMS ROIs were available to divide into folds, and the high AUCs indicated exceptional differentiation. The cancer model calculations utilized 4-fold analysis so had a distribution of AUCs for the validation data set. The PCa versus benign model had AUC mean = 0.93 and 95% CI (0.92, 0.93) for PZ and AUC mean = 0.92 and 95% CI (0.89, 0.94) for TZ. The high-grade versus low-grade model had mean = 0.70 and 95% CI (0.57, 0.82) for PZ and mean = 0.82 and 95% CI (0.81, 0.83) for TZ.

### 3.4. Cancer risk maps

Probability thresholds in the cancer maps of 70% (PCa versus benign) and 50% (high-grade PCa versus low grade PCa) were optimum for defining lesion masks. These thresholds minimized the number of outlier volumes and resulted in a cohort mean ratio of MRI to histopathology volume near one. Using these thresholds, cancer masks were generated, and MRI lesions assessed.

**Fig. 3.**
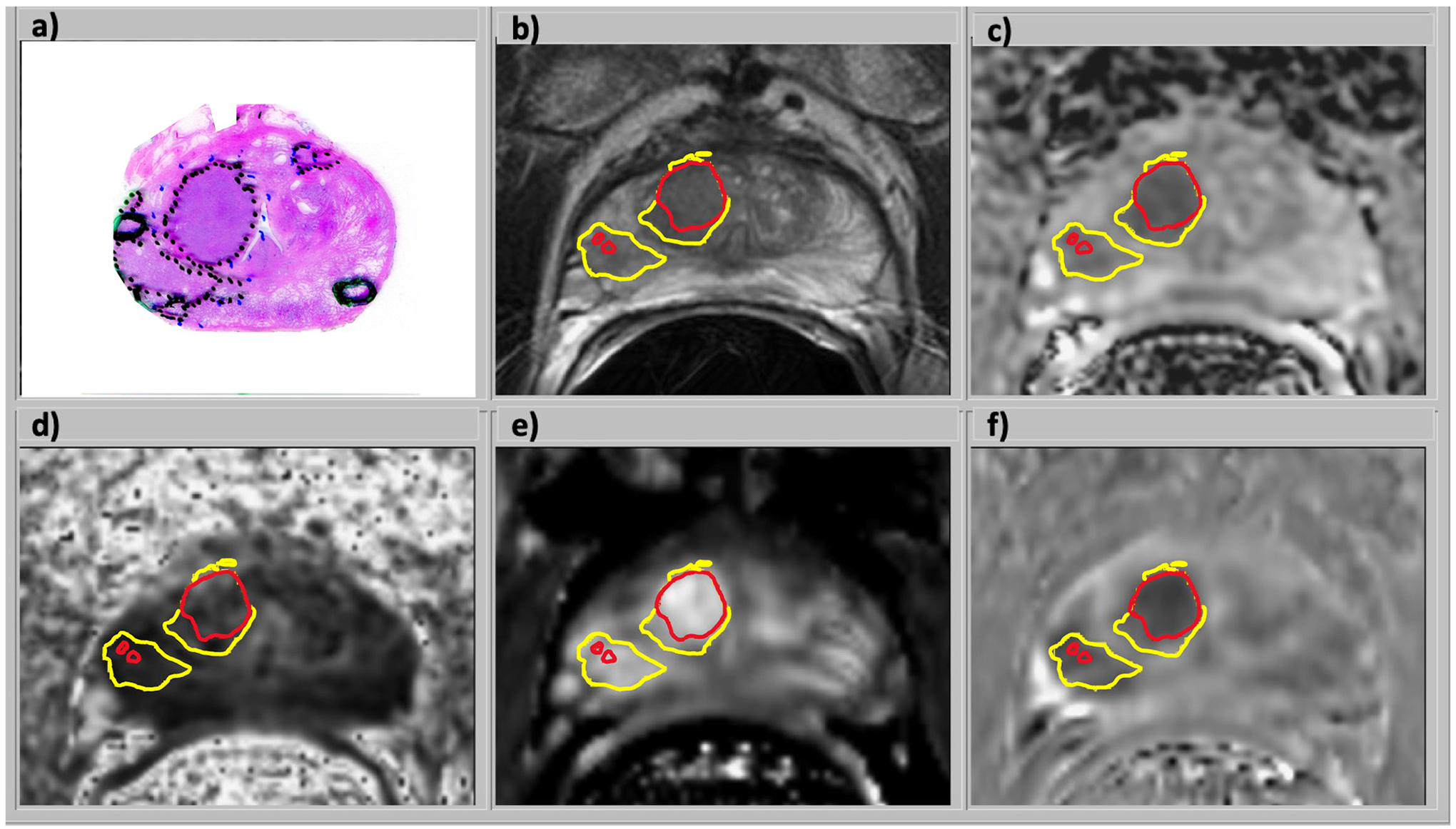
Images from a 70 year-old male with serum PSA of 9.8 ng/ml and GG3 prostate cancer who underwent radical prostatectomy: a) H&E stained histology specimen, b) coil-corrected T2-weighted FSE image, c) ADC map, d) fractional anisotropy map, e) DCE MRI enhancement slope, and f) DCE MRI washout slope. The mpMRI images were combined in a logistic regression model to generate the cancer risk maps in the TZ and the PZ. In this example, a combination of hypointense T2W, ADC, and DCE MRI washout plus hyperintense DCE MRI enhancement slope resulted in a high-risk region in the cancer maps and lesions identified by the outline of the cancer masks for all PCa (yellow) and aggressive PCa (red).

Fig. 3 is an example of histopathological and mpMR images, with outlines of the cancer risk map identified lesions. Various tissue types demonstrate different combinations of hyper- and hypo-intensities in the MR images. The cancer lesions exhibit hypointense T2W, ADC, and DCE MRI WO plus hyperintense DCE MRI ES which resulted in high-risk regions in the cancer map. Lesions were identified by the binary cancer mask.

**Fig. 4.**
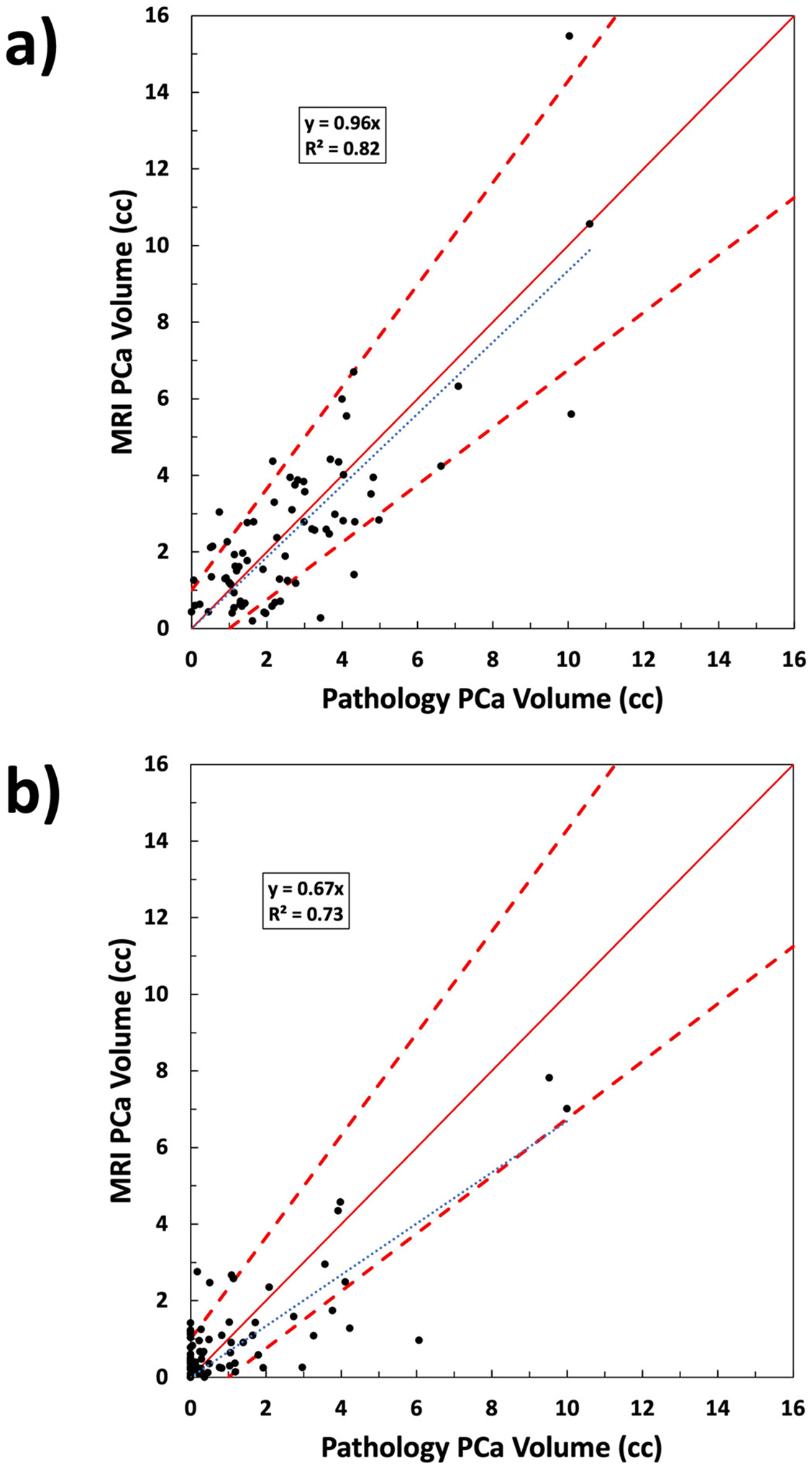
Prostate cancer volume comparison of MRI cancer map versus histopathology for a) all PCa and b) PCa with Gleason grade group 3–5. Cases with overestimated (underestimated) cancer volume are above (below) the solid red one-to-one line. Dashed bounding lines were defined to indicate outliers. Percentage of cases within the boundaries were 77% for a) and 75% for b).

**Fig. 5.**
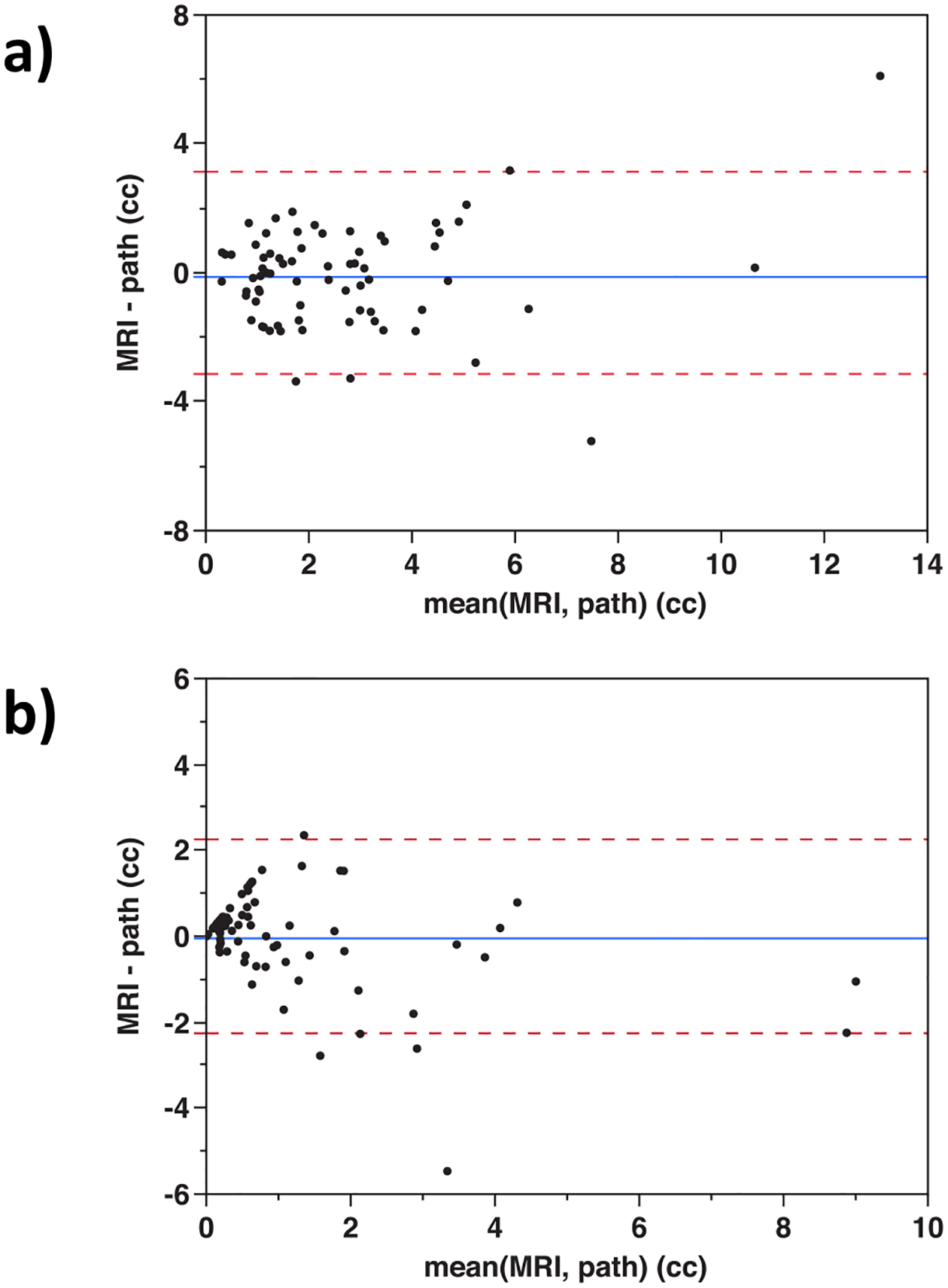
Bland-Altman plots of PCa volume for each case comparing MRI to pathology for a) total cancer volume and b) Gleason grade group 3–5 cancer volume.

The PCa volumes of MRI versus histopathology for each case are plotted in Fig. 4a for all cancer and Fig. 4b for high-grade cancer. Fig. 5a and b are Bland-Altman plots of difference between MRI and histopathology volumes. Characteristics of the MRI PCa volume distributions are summarized in Table 3.

MRI lesions were automatically localized in the prostate, then visually confirmed by comparison to histopathology (Fig. 1a and f). The MRI risk maps detected 135 / 150 of histopathological lesions >0.1 cc (TP = 135 and FN = 15) and falsely identified 32 regions as cancer (FP), yielding a sensitivity of 90% and a positive predictive value of 81%. The number of true negatives (TN) was 143 resulting in a specificity of 82%. The same analysis was applied to the subset of lesions below 1 cc in volume to better assess the method’s capability for small lesions. The MRI risk maps detected 75 / 85 of histopathological lesions between 0.1 cc and 1 cc (TP = 75 and FN = 10), and falsely identified 28 regions as cancer (FP), yielding a sensitivity of 88% and a positive predictive value of 73%.

The corresponding values for high-grade PCa were 51 / 65 HGROIs detected, FN = 14, FP = 39, TN = 68, sensitivity = 78%, and specificity = 64%. For HGROIs between 0.1 cc and 1 cc, the sensitivity was 86% and PPV was 48%. While the sensitivity for HGROIs was lower than for all PCa, almost all (62 / 65 (95%)) were identified as PCa of either low or high-grade.

**Fig. 6.**
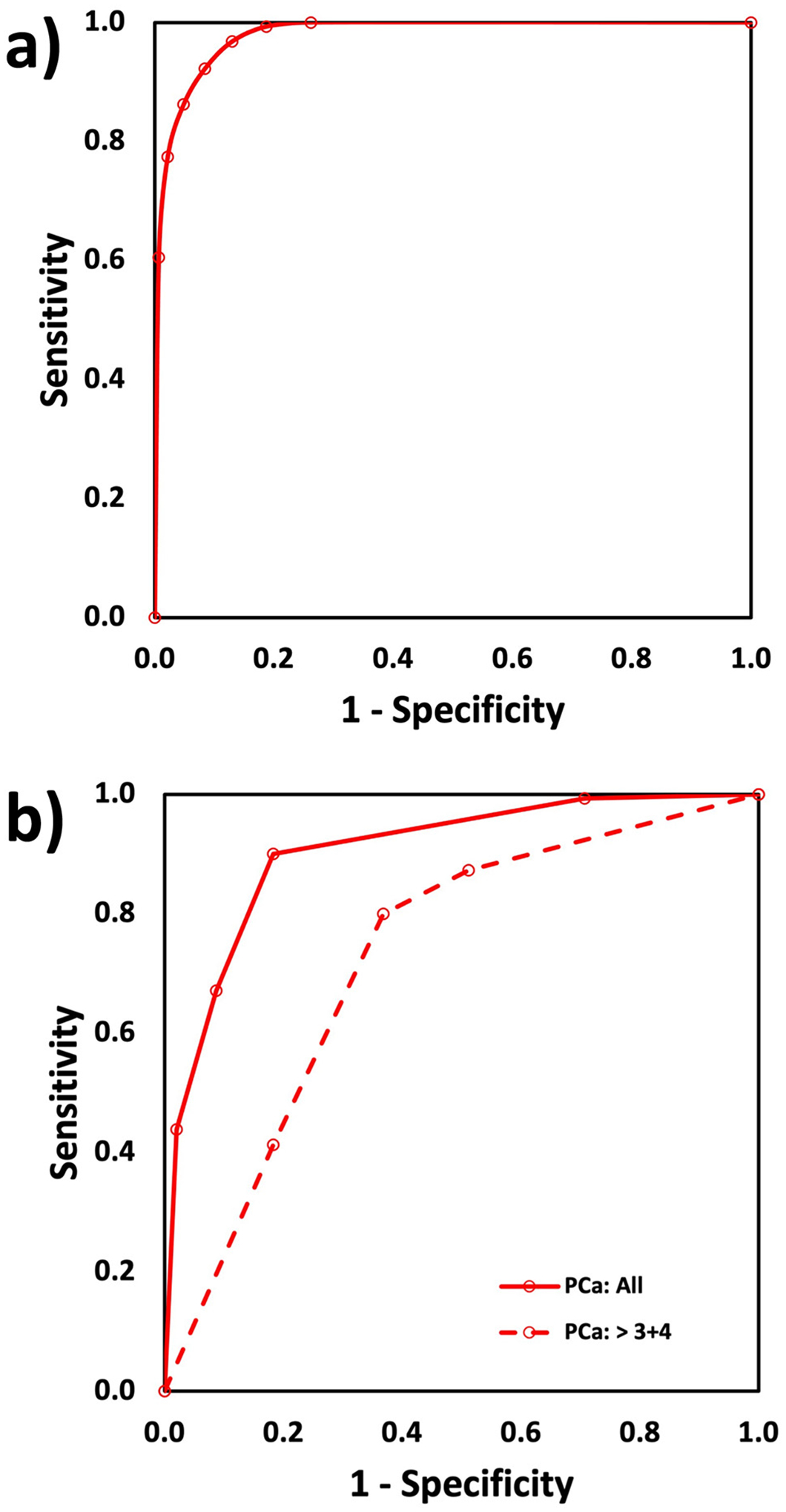
ROC curves for the PCa maps comparison of MRI versus histology: a) total volume of PCa and benign (AUC = 0.98) and b) PCa lesion analysis to identify lesions larger than 0.1 cc for all PCa vs benign (AUC = 0.91) and GG 3–5 vs GG < 3 (AUC = 0.73).

ROC curves were generated by performing analysis of MRI volumes versus histopathology volumes at several cancer probability thresholds and calculating the confusion matrix factors. The resulting curves are shown in Fig. 6. The ROC AUC for PCa volume versus benign tissue volume was 0.98 with confusion matrix as defined in Table 1. The ROC AUCs derived from the lesion analysis were 0.91 for all PCa and 0.73 for high-grade PCa with confusion matrix as defined in Table 2.

**Fig. 7.**
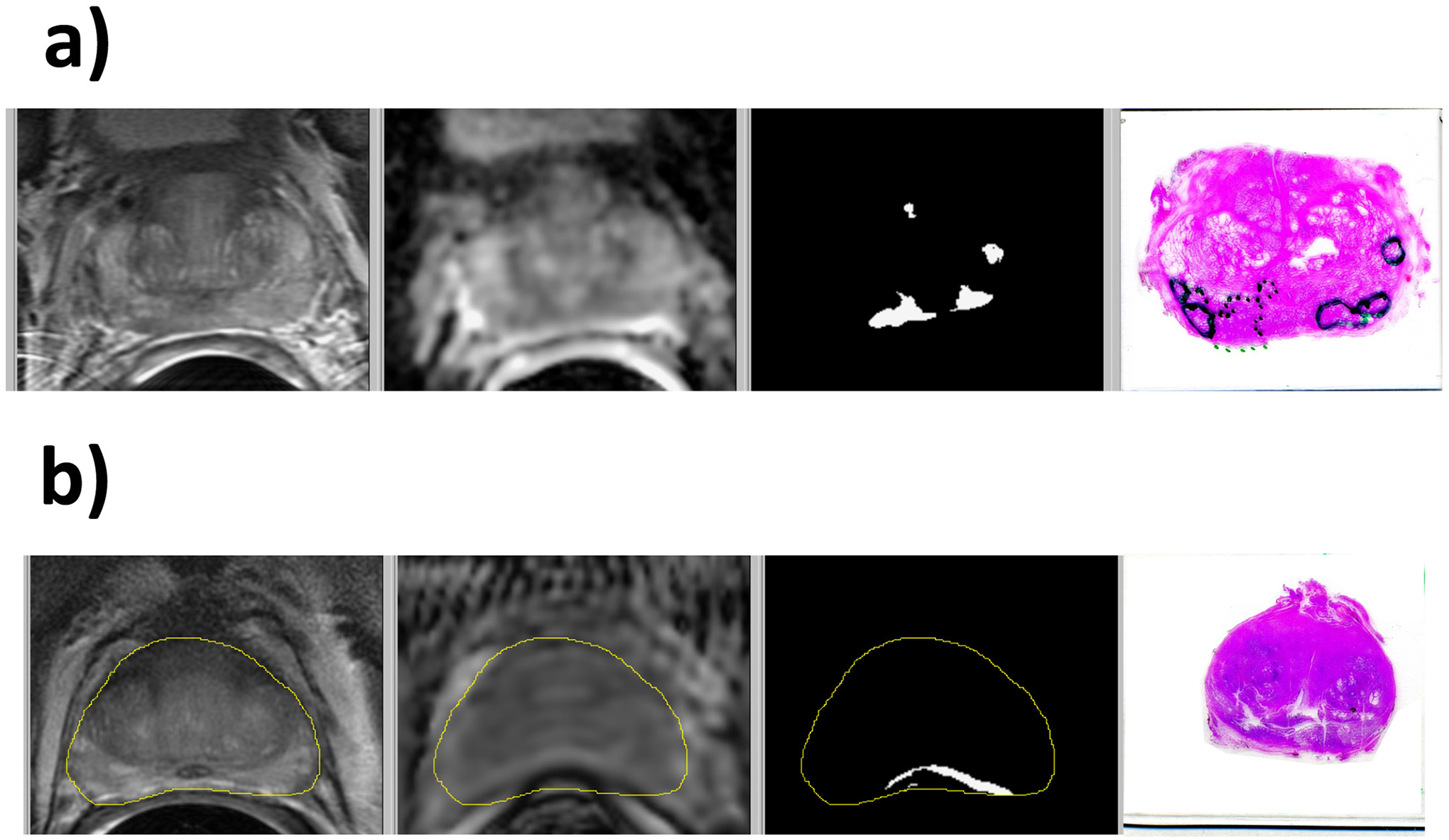
Examples of cases with outlier MRI PCa volumes: a) HGPIN resulting in false positive PCa in the left PZ and b) distortion of the ADC image causing false positive PCa near the rectum. The images are T2W, ADC, lesion mask, and histopathology. In the histology images, PCa is bounded by dotted lines, and HGPIN is bounded by solid lines.

False positive and cancer volume overestimates were evident when the prostate had significant volume of high grade prostatic intraepithelial neoplasia (HGPIN) (6% of cases) as shown in Fig. 7a. False negatives or false positives occurred when obvious distortion existed in the diffusion imaging (7% of cases) as shown in Fig. 7b. False negative and underestimates of cancer volume occurred when: 1) Images (3%) had anomalously low DCE MRI signals. 2) MRI parameters (4% of cases) caused risk to be near the cancer threshold.

## 4. Discussion

The MRI cancer risk maps had high sensitivity for detecting cancer while requiring a detection limit of 0.1 cc. Total PCa volumes, individual lesion volumes, and lesion locations tracked well with histopathology. The high AUC, 0.98, for total PCa volume vs benign volume is a necessary metric as a global indicator of PCa map accuracy; however, it is not sufficient. With a mean tumor burden of 9.5%, most of the prostate volumes were benign. Given this benign tissue dominance, high sensitivity was possible without significantly impacting specificity. Because of this, we also performed lesion assessment with location and volume analysis.

The lesion analysis achieved a sensitivity of 90% and a specificity of 81% despite the PCa volumes being small relative to the total prostate volumes. In the lesion analysis, most FP and FN were associated with lesions ≤ 1 cc. Nonetheless, analysis of the ≤ 1 cc subpopulation only reduced sensitivity to 88%. As evident in both the MRI versus histopathology volume plots and the Bland-Altman plots, the systematic error was small, and mean error did not trend with lesion size. Variability did not exhibit excessive outliers. The variability did appear to decrease for volumes below 1 cc. More data would be needed to confirm this trend, nonetheless, it is a desirable characteristic for variability to scale down with lesion size.

Assessing high-grade cancer was less accurate. However, the lower sensitivity was driven by differentiation of cancer grade not error in recognizing PCa versus benign. Use of two cancer models captured >95% of high-grade ROIs as cancerous. Having three categories (benign, low grade, and high-grade), provides more granularity in the reported metrics which may mitigate error due to prostate tissue variability and MRI scan artifacts.

These quantitative results could assist radiologists in their assessments by estimating lesion volume and grade. In addition to tumor load, the PCa masks can be utilized to extract mean MRI properties within cancer volumes. Quantities resulting from this method could be applied for population analysis of progression.

The automated characterization of PCa has been approached in various ways [11] including logistic regression [12,13], deep learning convolutional neural networks [14] [15], and risk scoring systems based on mpMRI factors [17]. The reported accuracy of these approaches depended on both the capability of the methods and the responses being categorized. The labeling granularity ranged from global (patient) to local (lesion). For example, some studies distinguished participants [27] and images (slice) [28] as having cancer or not. Others attempt to match the presence (or absence) and location of individual lesions [5,13,14,17,29,30]. The strictest objectives have been to resolve lesions of small size [31] while maintaining reasonable specificity. Meanwhile, the standard to determine accuracy also had several approaches. The ground truth may be MRI assessed by radiologist often with PI-RADS scoring [16,32,33], MRI confirmed by biopsy [15,27,28], or post-prostatectomy histopathology [12,17,30]. The histopathology data must be anatomically located on the MRI images but does not depend on specific MRI signatures at those locations. Finally, methods distinguish various tissue type combinations such as PCa from benign [12,16,28], clinically significant PCa from other tissues [13,30,31,33], or higher grade from lower grade PCa and benign [14,17,29,32].

These differences in study methodologies result in a wide range for accuracy metrics such as ROC AUC from 0.64 to 0.94 which make comparison difficult. However, this study did exhibit accuracies near the upper end of the range while utilizing relatively restrictive criteria. This included identification of individual lesions down to 0.1 cc, limits on minimum and maximum volume defined as a match between MRI and histopathology, distinguishing low- and high-grade PCa, and histopathology as the reference standard.

As shown in previous studies [18,19], mpMRI was necessary for improved AUC of the models. mpMRI had the ability to compensate in an individual when one parameter’s image was poor while other parameters were adequate. This was necessary to achieve correct mean volume for the whole population as well as properly differentiate lesions within each individual. Meanwhile, differences between model results and histopathology suggested root causes and potential improvements.

As noted in the results, several overestimates in PCa volume were caused by regions of HGPIN. HGPIN has been demonstrated to overlap PCa for MRI parameters DCE MRI ES and DCE MRI WO [18]. This overlap could be sufficient for the model to misidentify some HGPIN as PCa. As with the AFMS model, it may be possible to create a specific HGPIN versus PCa model to improve the lesion ROC AUC.

Distortion in DWI affected the shape and magnitude of ADC images. In some cases, the distortion caused misalignment of ROIs between the image types or shifted PCa volumes outside the prostate boundary. These effects tended to underestimate volumes of high cancer probability. Other distortions shifted the low ADC rectum into the prostate causing overestimation of PCa volume. Imaging with acquisitions less prone to artifact such as rFOV [22] benefits the risk maps. Diagnostics to detect rectum distortion of the DWI images versus the T2W images could be implemented to identify problematic results.

Despite DCE normalization, a small number of cases, 3%, were impacted by anomalous DCE signals. Potentially poor DCE cases could be identified by comparing results from models with and without DCE. Since DCE parameters tend to refine PCa map results but are not dominant, significantly different results with and without DCE may indicate suspect DCE images. These cases could be checked manually.

Some volumes of tissue had PCa probability near the threshold resulting in either over- or under- estimates of cancer. For example, the low MRI volume cases mentioned in the results all had >80% of their PCa as grade group 1. Low-grade PCa has MRI characteristics less distinct from benign [19]. Given MRI variability, some PCa voxels could have probability below threshold. Two modifications may improve cancer maps capability. First, additional parameters in the models may mitigate the overlap of MRI parameter distributions between benign and cancerous tissues improving tissue differentiation. Second, although ADC is an absolute quantity, it is affected by scan conditions (temperature, scan parameters, scanner calibration) [34–37]. To improve quantitative MRI capability, it may be necessary to calibrate ADC between scans. Lastly, mixture of benign tissues with the cancerous tissues could impact the strength of cancer signal.

It is evident in some lesion masks that parts of calculated MRI lesions extend beyond what might be expected from manual inspection. Results such as this occur because of errors in image mis-registration, prostate segmentation, and DWI distortion. These errors combined with anatomical structures having low ADC, such as the urethra and surrounding stroma or tissue just outside the prostate capsule, could lead to false positive indications of PCa. By applying the AFMS mask, these types of errors were found to be mitigated. The high AUC for the AFMS model was achieved with MRI parameter coefficients different than those for the other models as shown in the supplemental information. FA has a significant contribution in distinguishing AFMS [18].

This study was limited by the semi-automated method of matching MRI lesions to pathology lesions for lesion detection assessment. An alternative, fully automated method would involve creating a digitized lesion mask from the pathology images and performing morphological, nonlinear registration of the pathology images to the MRI T2W images [12]. Then categorization of lesion overlap could be performed with dice coefficient analysis between MRI and pathology lesions. However, error would remain inherent in the nonlinear registration procedure which may or may not be less than the manual method used here. Given distortions in both MRI images and pathology samples, it may be unfeasible to expect automated matching of lesions with sizes down to 0.1 cc. In fact, Metzger, et al. [12] performed ROC analysis over the whole population of prostate voxels not individual lesions. Because of these limitations in automated matching, the manual matching method was applied for this study. Meanwhile, the manual method still screened for MRI lesions co-located but significantly larger (FP) or smaller (FN) than histopathology lesions.

A second limitation of this study was uncorrected distortion in DWI images, which may have led to errors in cancer localization. While non-rigid registrations may have improved the matching of tissues, signal pile-up or loss would still affect cancer map estimations. Corrections of these artifacts would require more involved methodology. While these artifacts were present in the study, they were not so prevalent to detract from this study’s overall performance in detecting, localizing, and quantifying cancer volumes in this cohort.

## 5. Conclusion

This study showed the feasibility of MRI generated cancer risk maps, created from pre-prostatectomy, mpMRI images validated with histopathology, to detect PCa lesions >0.1 cc and to quantify volume and location of cancer. Root causes for over and underestimation of cancer volumes were identified. Improved capability to quantify PCa could aid physicians and patients in treatment decisions, targeting biopsy, planning focal therapy, and predicting progression.

## Supplementary Material

1

## Figures and Tables

**Table 1:** Definition of factors to determine specificity and sensitivity for tumor burden.

Factor	Definition
True Positive (TP) volume	minimum (MRI volume, pathology volume)
False Positive (FP) volume	maximum (MRI volume – pathology volume, 0)
True Negative (TN) volume	prostate volume - maximum (MRI volume, pathology volume)
False Negative (FN) volume	maximum (pathology volume – MRI volume, 0)

**Table 2:** Definition of factors to determine specificity and sensitivity for individual lesions.

Factor	Definition
True Positive (TP)	MRI and histology lesion in same location and *MRI lesion vol* > 0.1 cc and *histology lesion vol* > 0.1 cc and *MRI lesion vol* ≥ 0.75 • *histology lesion vol* − 1 cc
False Positive (FP)	MRI lesion present but no histology lesion present or *MRI lesion vol* > 1.33 • *histology lesion vol* + 1 cc
True Negative (TN)	*MRI lesion vol* ≤ 1.33 • *histology lesion vol* + 1 cc
False Negative (FN)	Histology lesion present but no MRI lesion present and *histology lesion vol* > 0.1 cc or *MRI lesion vol* < 0.75 • *histology lesion vol* − 1 cc
Not Applicable (NA)	*MRI lesion vol* < 0.1 cc and *histology lesion vol* < 0.1 cc

**Table 3:** Cohort characteristics of PCa identified by pathology (top) and MRI (bottom).

Characteristic		Value
Number of lesions		269
Number of lesions with volume > 0.1 cc		150
Volume (mean, STD)		(0.55 cc, 0.98 cc)
Diameter median		6 mm
Diameter minimum		2 mm
Tumor burden (mean, STD)		(2.7 cc, 2.2 cc)
Tumor burden / prostate volume (mean, STD)		(0.10, 0.07)
PCa type	All cancer	High-grade cancer
Number of participants	73	73
MRI volume outliers (see Fig. 4)	17 (23%)	18 (25%)
Bland-Altman difference (MRI – path)		
All cases (mean, STD)	(−0.12 cc, 1.59 cc)	(−0.06 cc, 1.16 cc)
Cases volume < 1 cc (mean, STD)	(0.009 cc, 0.90 cc)	(0.24 cc, 0.55 cc)
Cases volume > 1 cc (mean, STD)	(−0.14 cc, 1.69 cc)	(−0.69 cc, 1.76 cc)

## Abbreviations:

ADC: apparent diffusion coefficient
AFMS: anterior fibromuscular stroma
AUC: area under the receiver operating characteristics curve
DCE MRI: dynamic contrast-enhanced magnetic resonance imaging
DCE MRI PE: dynamic contrast-enhanced peak enhancement
DCE MRI ES: dynamic contrast-enhanced enhancement slope
DCE MRI WO: dynamic contrast-enhanced washout slope
DWI: diffusion weighted imaging
FSE: Fast Spin Echo
GS: Gleason Score
GG: Gleason Grade Group
HGPIN: high grade prostatic intraepithelial neoplasia
HGROI: high grade region of interest
K^trans^: volume transfer constant
LR: logistic regression
MP: multiparametric
PCa: prostate cancer
PZ: peripheral zone
ROI: region of interest
TZ: transition zone
