# Supplementary material for: Prostate cancer lesion detection, volume quantification and high-grade cancer differentiation using cancer risk maps derived from multiparametric MRI with histopathology as the reference standard
Source: Magn Reson Imaging. Author manuscript; Available in PMC 2024 Jul 8. (PMC11229728; doi:10.1016/j.mri.2023.01.006)
Supplement: 1 [file NIHMS2004256-supplement-1.docx]

**Supplemental Information:**

Table S1: Counts for the tissue regions of interest (ROI) used to generate the logistic regression models for PCa vs benign, PCa all vs PCa high-grade, and PCa vs AFMS

| Tissue Type | PCa | Benign | AFMS | Total |
| --- | --- | --- | --- | --- |
| Number of ROIs in TZ | 113 | 444 | 111 | 668 |
| Number of ROIs in PZ | 284 | 572 | 0 | 856 |
| Number of ROIs in total | 397 | 1016 | 111 | 1524 |

The identified tissues included: PCa by grade, AFMS, atrophy, BPH, cystic atrophy, inflammation, normal, stroma, HGPIN. The counts in the benign column do not include AFMS.

Table S2: Coefficients for logistic regression fit (PCa vs benign)

|  |  | PCa model | | AFMS model | |
| --- | --- | --- | --- | --- | --- |
| Parameter | Fit coefficient | PZ | TZ | PZ | TZ |
| Constant | A | 6.755 | 5.665 | -2.944 | 1.033 |
| T2W_norm_ | B_1_ | -0.162 | 2.788 | 0 | 0 |
| ADC | B_2_ (10^6^ s / mm^2^) | -0.00582 | -0.00617 | 0 | 0 |
| ES_norm_ | B_3_ (min / %) | 0.0178 | 0.0078 | -0.0619 | -0.0756 |
| WO_norm_ | B_4_ (min / %) | -0.0538 | 0 | 0 | 0 |
| FA | B_5_ (1 / %) | 0 | 0 | 27.57 | 17.55 |
| (T2W_norm_)^2^ | C_1_ | 0 | -0.847 | 0 | 0 |
| AUC |  | 0.924 | 0.914 | 0.996 | 0.986 |

|  |  | PCa PZ model | | | |
| --- | --- | --- | --- | --- | --- |
| Parameter | Fit coefficient | Fold 1 | Fold 2 | Fold 3 | Fold 4 |
| Constant | A | 7.642 | 5.966 | 4.041 | 7.426 |
| T2W_norm_ | B_1_ | -0.152 | -0.201 | 1.681 | -0.300 |
| ADC | B_2_ (10^6^ s / mm^2^) | -0.00631 | -0.00520 | -0.00615 | -0.00596 |
| PE_norm_ | B_3_ (min / %) | 0.0178 | 0.0189 | 0.0199 | 0.0165 |
| WO_norm_ | B_4_ (min / %) | -0.125 | -0.0492 | 0 | -0.0724 |
| FA | B_5_ (1 / %) | 0 | 0 | 0 | 0 |
| (T2W_norm_)^2^ | C_1_ | 0 | 0 | -0.273 | 0 |
| AUC training | | 0.930 | 0.912 | 0.932 | 0.927 |
| AUC validation | | 0.903 | 0.958 | 0.898 | 0.918 |
| AUC training | | Mean = 0.925 95% CI = (0.916, 0.934) | | | |
| AUC validation | | Mean = 0.919 95% CI = (0.893, 0.946) | | | |

|  |  | PCa TZ model | | | |
| --- | --- | --- | --- | --- | --- |
| Parameter | Fit coefficient | Fold 1 | Fold 2 | Fold 3 | Fold 4 |
| Constant | A | 4.673 | 5.966 | 4.041 | 7.426 |
| T2W_norm_ | B_1_ | 3.353 | -1.468 | 3.649 | 4.319 |
| ADC | B_2_ (10^6^ s / mm^2^) | -0.00581 | -0.00606 | -0.00842 | -0.00551 |
| PE_norm_ | B_3_ (min / %) | 0.00753 | 0.0126 | 0.00722 | 0.00528 |
| WO_norm_ | B_4_ (min / %) | 0 | 0 | 0 | 0 |
| FA | B_5_ (1 / %) | 0 | 0 | 0 | 0 |
| (T2W_norm_)^2^ | C_1_ | -0.958 | 0 | -1.092 | -1.103 |
| AUC training | | 0.908 | 0.911 | 0.948 | 0.897 |
| AUC validation | | 0.964 | 0.913 | 0.829 | 0.967 |
| AUC training | | Mean = 0.916 95% CI = (0.894, 0.938) | | | |
| AUC validation | | Mean = 0.918 95% CI = (0.855, 0.981) | | | |

Table S3: Coefficients for logistic regression fit (Grade groups 1&2 vs Grade groups 3 – 5)

|  |  | PCa model | |
| --- | --- | --- | --- |
| Parameter | Fit coefficient | PZ | TZ |
| Constant | A | 4.614 | -3.183 |
| T2W_norm_ | B_1_ | -0.819 | 0 |
| ADC | B_2_ (10^6^ s / mm^2^) | -0.00229 | 0 |
| ES_norm_ | B_3_ (min / %) | 0.0 | 0.0166 |
| WO_norm_ | B_4_ (min / %) | -0.241 | -0.418 |
| FA | B_5_ (1 / %) | 0 | 0 |
| (T2W_norm_)^2^ | C_1_ | 0 | 0 |
| AUC |  | 0.781 | 0.828 |

|  |  | PCa PZ model | | | |
| --- | --- | --- | --- | --- | --- |
| Parameter | Fit coefficient | Fold 1 | Fold 2 | Fold 3 | Fold 4 |
| Constant | A | 3.219 | 2.920 | 3.118 | 6.452 |
| T2W_norm_ | B_1_ | -1.070 | -0.524 | -0.710 | -0.824 |
| ADC | B_2_ (10^6^ s / mm^2^) | -0.00118 | -0.00148 | -0.00206 | -0.00361 |
| PE_norm_ | B_3_ (min / %) | 0.00444 | 0 | 0.00437 | 0 |
| WO_norm_ | B_4_ (min / %) | -0.164 | -0.246 | -0.282 | -0.221 |
| FA | B_5_ (1 / %) | 0 | 0 | 0 | 0 |
| (T2W_norm_)^2^ | C_1_ | 0 | 0 | 0 | 0 |
| AUC training | | 0.753 | 0.729 | 0.803 | 0.810 |
| AUC validation | | 0.748 | 0.854 | 0.597 | 0.592 |
| AUC training | | Mean = 0.774 95% CI = (0.735, 0.812) | | | |
| AUC validation | | Mean = 0.698 95% CI = (0.574, 0.822) | | | |

|  |  | PCa TZ model | | | |
| --- | --- | --- | --- | --- | --- |
| Parameter | Fit coefficient | Fold 1 | Fold 2 | Fold 3 | Fold 4 |
| Constant | A | -2.251 | -3.981 | -3.135 | -2.993 |
| T2W_norm_ | B_1_ | 0 | 0 | 0 | 0 |
| ADC | B_2_ (10^6^ s / mm^2^) | 0 | 0 | 0 | 0 |
| PE_norm_ | B_3_ (min / %) | 0.0109 | 0.0223 | 0.0141 | 0.0146 |
| WO_norm_ | B_4_ (min / %) | -0.481 | -0.305 | -0.495 | -0.418 |
| FA | B_5_ (1 / %) | 0 | 0 | 0 | 0 |
| (T2W_norm_)^2^ | C_1_ | 0 | 0 | 0 | 0 |
| AUC training | | 0.822 | 0.828 | 0.824 | 0.809 |
| AUC validation | | 0.875 | 0.830 | 0.831 | 0.880 |
| AUC training | | Mean = 0.821 95% CI = (0.812, 0.829) | | | |
| AUC validation | | Mean = 0.854 95% CI = (0.827, 0.881) | | | |


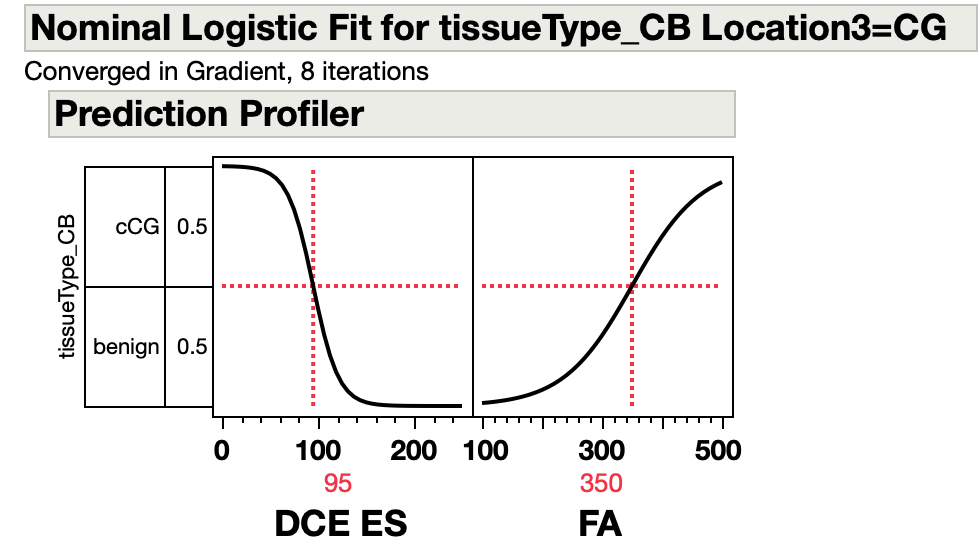

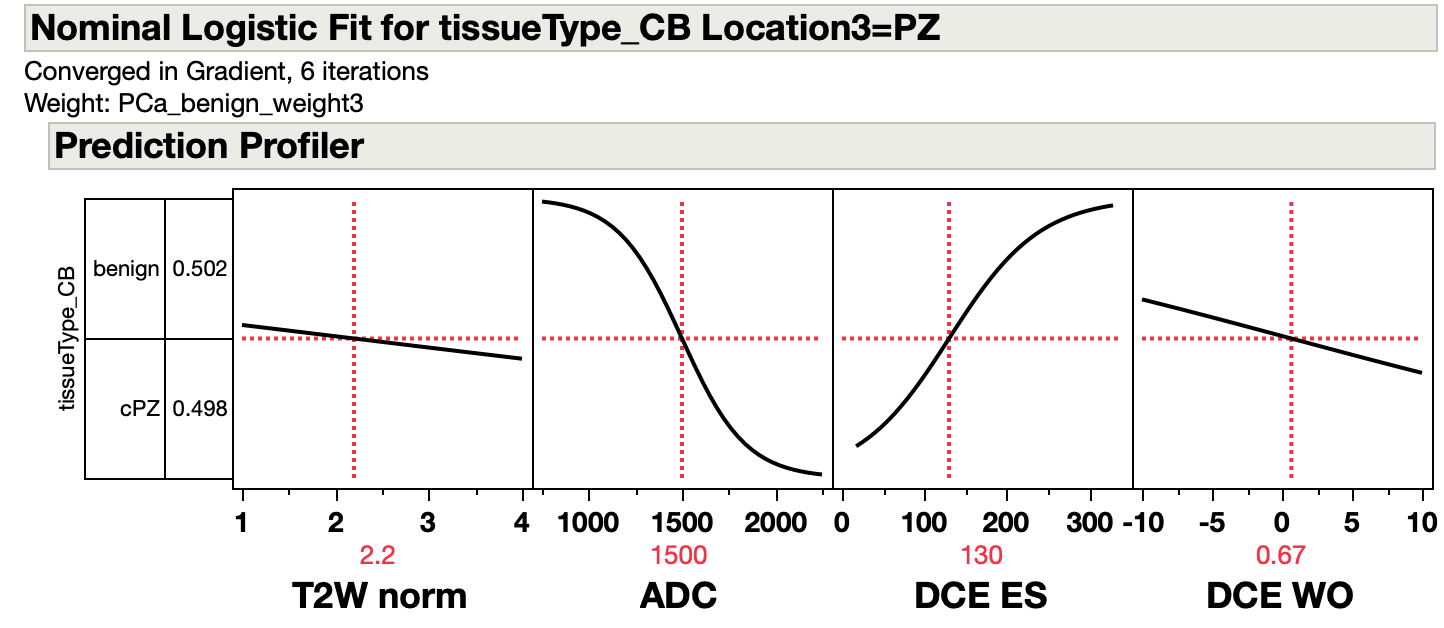


**0**

**1**

**1**

**0**

**1**

**b)**

**PCa**

**prob**

**0.5**

**a)**

**AFMS**

**prob**

**0.5**


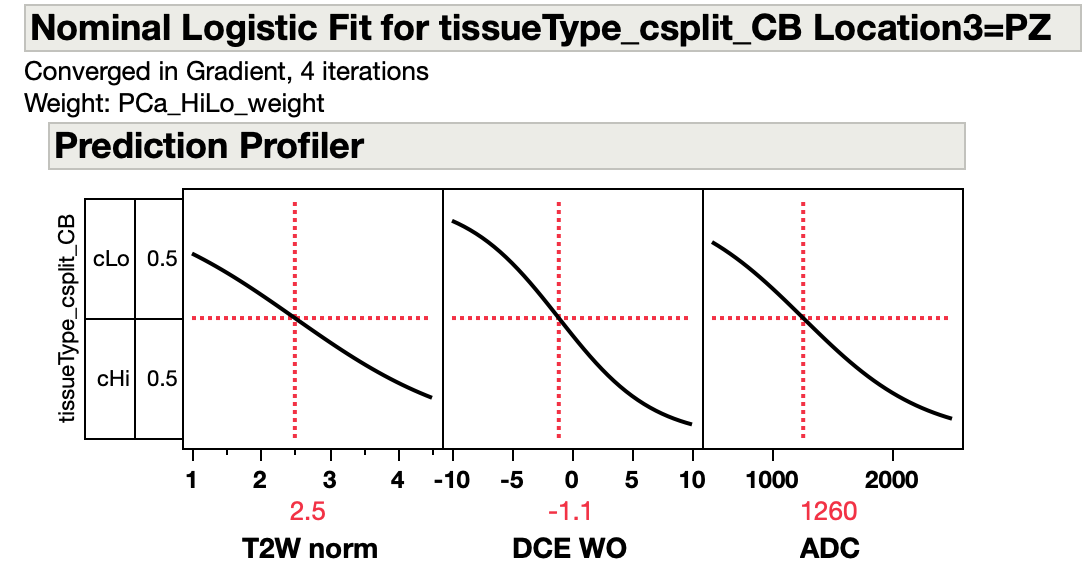


**0**

**c)**

**PCa Hi**

**prob**

**0.5**

Figure S1: Profiler plots of the logistic regression probability models with cursors set at parameters to have probability of 50%: a) AFMS vs PCa in the TZ, b) PCa versus benign in the PZ, and c) PCa high grade vs PCa low grade in the PZ.
